# History of a Dewdrop

**Published:** 1889-04

**Authors:** Samuel Brazier


### HISTORY OF A DEWDROP.

BY SAMUEL BRAZIER, IN POPULAR SCIENCE NEWS.

In any of the fairy scenes of nature what can be found more innocent, more beautiful and pure, than a morning dewdrop glistening in the cup of a flower? Sometimes from a distance of several yards away it reveals its presence by the bright ray of light it has just received from the sun, and flashes out again. You approach the glistening little object and try to look into its tiny depths. From one point of view it is dark, almost black; from another it is so bright that you cannot look into it at all: its reflected ray is as pure and intense as that of the most brilliant diamond. Then, looked at in a different direction, it is clear and transparent, looking so cool and beautiful. There it is, trying to be a perfect little globe, which it would be if it were not for the pressure of its weight on the petals of the flower. But see, while you look into it and admire it, it gradually becomes less and less, till, kissed by the rays of the morning sun, it vanishes away. The heat contained in the rays of light that were passing through it was sufficient to overcome the mutual attraction of the molecules of water of which it was composed, and alter its form from liquid to vapor. It has again become invisible moisture, and floated away in the air, from which, during the night, when the air was cooler, and therefore more contracted and unable to hold so much moisture, the little dewdrop was squeezed out into the cool cup of the flower. Where did it come from and how did it get into the night air, ready to be deposited as dew? And where is it gone to now, and what changes will it undergo? The dewdrop has become invisible, but is it lost? No; no atom of matter can ever be lost or destroyed, or cease to be of use. It is as impossible to annihilate an atom of matter as to create one. The liquid gem that glistened in the rays of the morning sun disappears from view, but in the form of invisible vapor it may be inhaled by the breathing leaves of plants or trees, or it may rise to become again visible in the floating cloud, and, by reflecting and refracting the solar rays, may help to paint the glowing sunset. To-morrow, it may descend to the earth again as rain, to be drunk up by the roots of some thirsty plant, or serve to refresh some happy singing-bird, so entering into the composition of animal or vegetable form; or it may trickle through some crevice in the ground into the reservoir beneath, and, mingling with the streams that flow in the interior of the earth, may ooze out again in some cool spring, join the stream of the valley, rippling and dancing on its way to swell the volume of the river, dash down the waterfall, and then emerge into the ocean, there to be again evaporated by the sun, or sparkle on the crest of the wave that the tide rolls in upon the shore; or the dewdrop that to-day is exhaled from the heart of a flower and rises to become cloud, may be carried by winds hundreds of miles away into colder regions, and fall in the form of hail or snow. It may roll down in the avalanche into the valley, or, falling as a snow-flake on some lofty mountain peak, it may form part of the glacier or ice-river that glides slowly down the valley into the sea. There it dips down into the water, but, ice being lighter than water, it tends to rise to the surface, and occasionally, immense blocks of ice are broken off and float away. These are the icebergs, which, floating into warmer latitudes, gradually melt and mingle with the waters of the ocean.

But through all these various transformations the dewdrop continues to be water. Its changes from solid to liquid and from liquid to aeriform are caused by differences of temperature. These are physical changes.

It may, however, undergo chemical changes, by which it may cease to be water in any form. Water is composed of two invisible gases,—hydrogen and oxygen. Oxygen has a strong affinity for iron. If the dewdrop is deposited on iron instead of a leaf or flower, the iron robs the water of some of the atoms of oxygen, and lets the hydrogen go free. This union of iron with the oxygen of the water forms the thin film of rust, or red oxide of iron, observable on iron that has been exposed to dew or rain.

In other ways also water may be decomposed, and its oxygen or hydrogen enter into the composition of other substances. So the dew-drop we admire to-day may continue to be water in the form of vapor, or liquid, or solid ice, or snow, for thousands of ages; or it may be decomposed in a few hours, and cease to be water in any of its forms. Nor can we tell how long the dew-drop has been water. It might have been water in some form for ages, or it might have been created by the decomposition of some other substance a few hours ago. Coal-gas, which is used to light the streets, contains hydrogen, which in burning unites with the oxygen of the air to form water, which passes away as invisible vapor. The watery vapor, therefore, which has been condensed to form the dew-drop, may have been produced during the night by the burning of the gas in the streets. The cool dewdrop might be born of the burning flame. Its atoms might have been sleeping for ages, hundreds of yards below the present surface of the earth, in the coal-bed composed of the vegetation of some forest of the ancient world. Or they might have entered into the formation of some limestone rock on which the beds of coal rested, or the more ancient granite rock, still lower down in the earth. There each tiny atom might have done its best for ages to uphold the mighty mountain above it, till, rent by earthquakes or melted by internal fires, and poured forth from the crater of some volcano, it was again brought under the influences of sun and air, of heat and light, and moisture and frost, and subjected to physical and chemical changes, till it came to sparkle in the morning dewdrop.

But, whatever wondrous transformations the dewdrop may have passed through, we know that it existed even when there was no cooling dew, nor thirsty flowers, nor forests, nor fields, nor lofty mountains, nor pleasant valleys, nor rolling rivers, nor widespread ocean, nor solid land; when the earth and all it now contains was vapor. "The earth was without form and void." During ages longer than we can count, this great mass of vapor cooled down till some parts became solid; some still remain fluid, and others gaseous, as the air that surrounds the globe.

From a shapeless mass of vapor it has been slowly transformed into the beautiful world it is to-day, where flowers bloom, and birds sing, and children play. During all those long ages each atom in the dewdrop has existed somewhere in some form. Each atom of hydrogen or oxygen or each molecule of water has done its duty and served some useful purpose. So it will continue to do in the countless ages to come. It will never be destroyed nor lost. It will be useful or beautiful somewhere forever.

How many questions must be asked and answered, how much and how varied information must be obtained, before we can understand the history and mystery of a dewdrop! The tiniest object in nature may suggest inquiries which may lead us into delightful paths in the broad fields of knowledge. A flower, a grain of sand, the commonest pebble you can pick up in the road, have each a wonderful story to unfold to those who take the pains to inquire and learn. So with this drop of dew. If we would follow out all the inquiries it suggests, we should have to study those same laws of nature that made the dewdrop round and the earth a sphere, and control the motions of planets and stars. We must learn how the dewdrop differs from a raindrop, or the hailstone or snowflake, or from cloud and fog and mist, into all of which it may be transformed. It is one form of water. Water has compressibility, specific gravity, and other physical and chemical qualities which enable it to perform very important uses in the life of animals and plants, and in the formation of wells in the earth. On the surface of the earth water occurs as lakes, streams, rivers, and the great ocean. So we should have to learn the influences of the ocean, lakes, and rivers on the climate of the globe, and their uses to man; and watersheds and hydraulic engines would demand attention. Then, water occurs in the form of rivers and lakes underground, where it is an active agent in causing earthquakes and forming caves. It reappears as springs which are sometimes fresh, sometimes salt. Some springs contain large quantities of mineral matter; some are cold, some are hot. Intermittent springs, geysers, artesian wells, would form subjects of inquiry. A whole chapter is required even to indicate the various subjects of study in many different sciences that must be pursued, and a lifetime may be spent in acquiring the knowledge that is necessary to enable us to understand the history and mystery of a dewdrop.

